# Isolated congenital absence of lower lateral cartilages: A four cases report

**DOI:** 10.1016/j.jpra.2018.09.003

**Published:** 2018-10-06

**Authors:** Jaafer H. Zainy

**Affiliations:** F.I.C.M.S, consultant plastic surgeon, Arab board of health specializations,Baghdad, Iraq

**Keywords:** Congenital nasal deformities, Lower lateral cartilages, Rhinoplasty, Septorhinoplasty, Nasal reconstruction, Absent nasal cartilages

## Abstract

**Background:**

Although isolated congenital absence of lower lateral cartilages are rare deformities, they deserve special attention of plastic surgeons performing rhinoplasty as these anomalies may pass unnoticed by the patient and the plastic surgeon; until confronting them during surgery.

**Methods:**

From August 2017 to December 2017, four cases of segmental loss of lower lateral cartilages were discovered accidentally during open primary septorhinoplasty of a total series of 250 cases. They were unnoticed by the surgeon preoperatively and the patients were unaware of these problems cosmetically or functionally. There was no history of trauma, major infection or any intervention in all four patients. Reconstruction of lower lateral cartilages was done with septal cartilage graft as part of the septorhinoplasty procedure.

**Results:**

All surgeries were uneventful postoperatively and all patients were satisfied with the results.

**Conclusion:**

On examining the patients for the rhinoplasty, one should be aware of subtle signs that may signify an underlying deformity in order for the patient to be informed about the complexity of the technique and for the surgeon to be prepared for the requirement of surgery.

## Introduction

The prevalence of congenital nasal anomalies is about 1 in 20,000 to 1 in 40,000 live births.^1^The majority have hypoplastic elements and usually genetic or syndromic.^2^ Isolated non-syndromic congenital nasal anomalies are rare.^3^ The patients may present for cosmetic or functional causes and usually they are not aware of this specific deformity. The use of drugs during pregnancy may represent an attributing factor for this type of deformity such as Carbimazol.^4^ Different cartilaginous sources have been used for reconstruction such as lower lateral cartilage, conchal cartilage, septal cartilage or even dorsal nasal hump cartilage.^1^, ^4^^,^^5^, ^7^

## Material and methods

From the period August 2017 to December 2017, out of total of 250 patients, a consecutive series of four cases of different forms, degrees and locations of isolated non-syndromic congenital segmental absences of nasal lower lateral cartilages, which were accidentally discovered, were operated for primary open septorhinoplasty. All cases were managed by reconstruction of the defected cartilages by septal cartilage graft as a part of the total septorhinoplasty. The anthropological data and the local findings are presented in Table 1.

**Table 1:** Details of patient's information, signs and symptoms.

Case	Sex	Age	Side	Location and extent	Signs	Symptoms
No.				of the defect		
1.	Female	20	Lt.	Medial & middle crura	Smaller nostril	Nill
2.	Female	30	Lt.	Almost total absence of LLC	Smaller nostril, deeper alar crease, furrow at soft triangle	Nill
3.	Female	25	Rt.	Middle & medial crura	Depressed dome, Smaller nostril	Nill
4.	Male	31	Lt.	Middle & lateral crura	Deep alar crease, flatdome, furrow at soft triangle, horizontally oriented nostril	Nill

## Results

All patients had an uneventful course postoperatively and all of them were satisfied with the results. Table 1 shows the patients’ information and findings.

## Discussion

**Figure 1:**
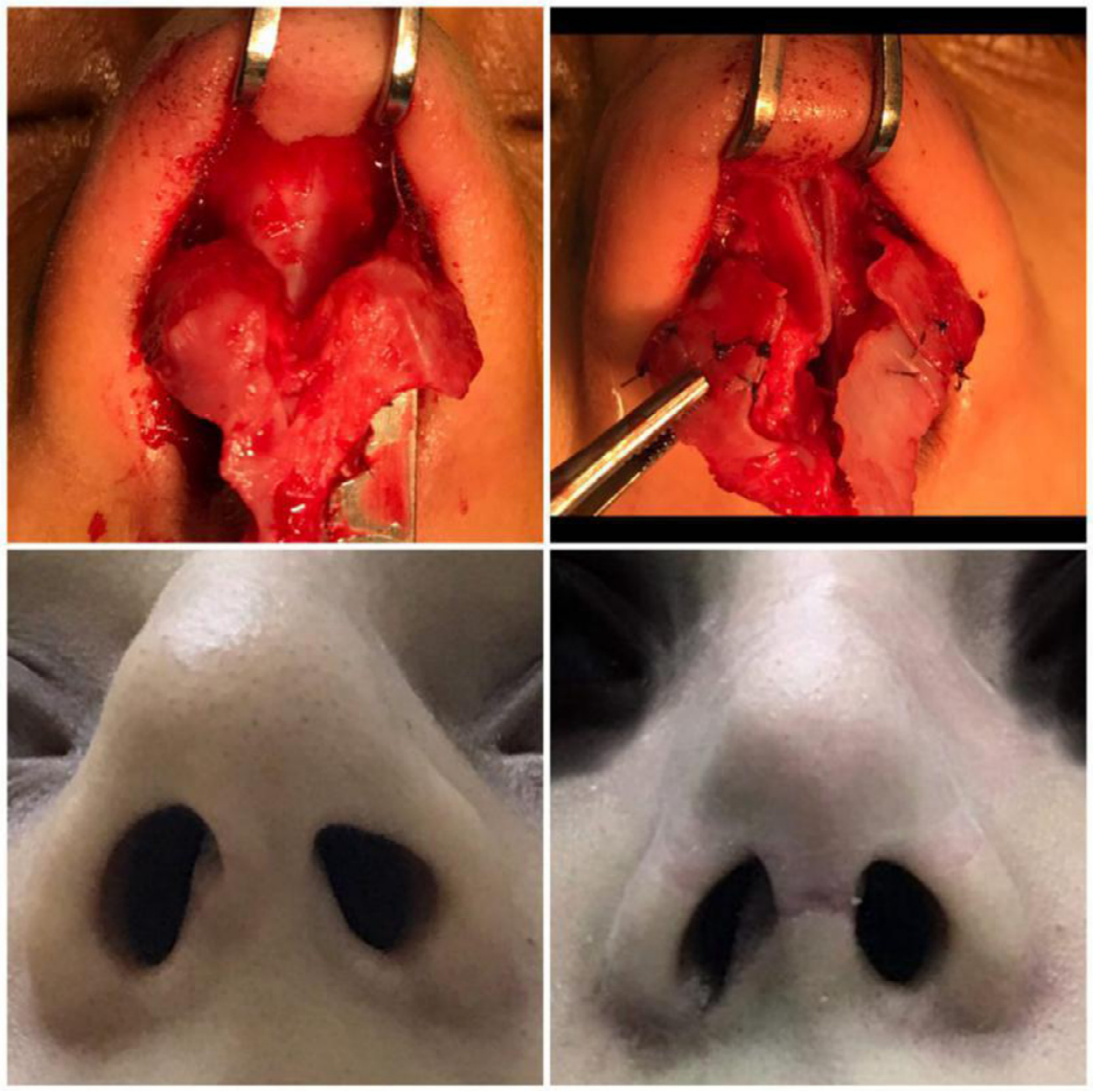
Case No. (1): Upper left; preoperative findings, Upper right; reconstruction of the defect Lower left; preoperative finding, Lower right; postoperative result.

**Figure 2:**
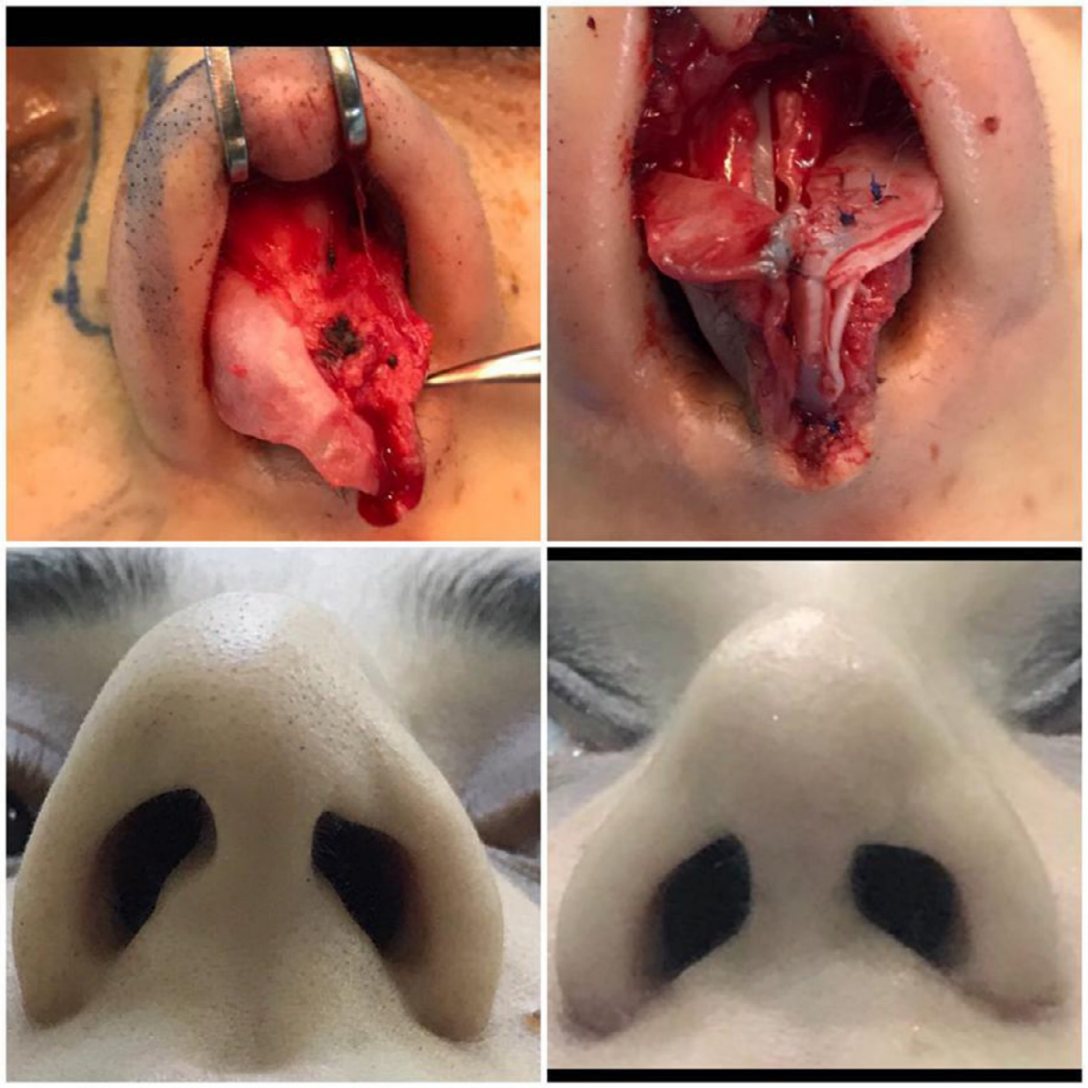
Case No. (2): Upper left; preoperative finding, Upper right; reconstruction of the defect, lower left; preoperative finding, Lower right; postoperative result.

**Figure 3:**
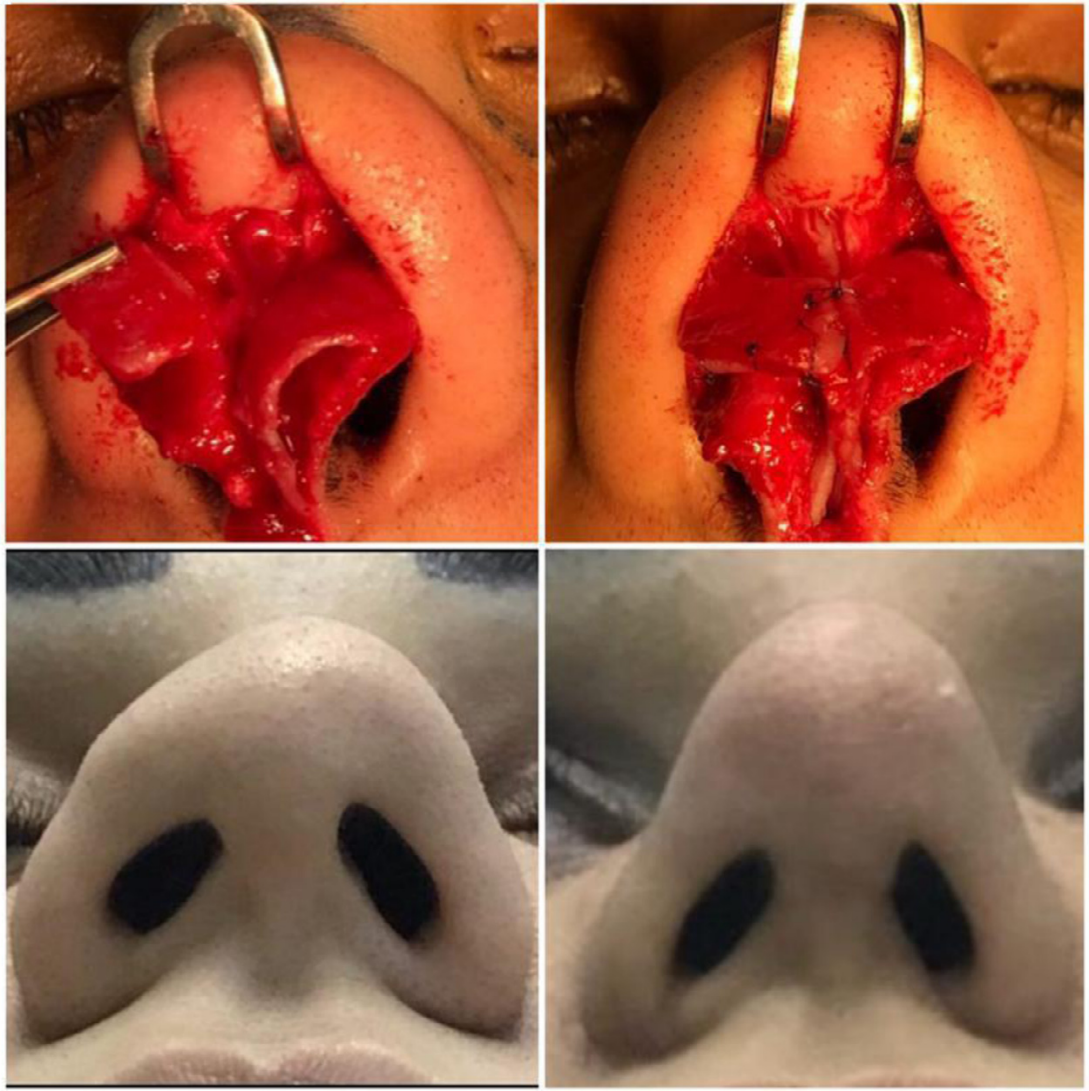
Case No. (3): Upper left; preoperative finding, Upper right; reconstruction of the defect, Lower left; preoperative finding, Lower right; postoperative result.

**Figure 4:**
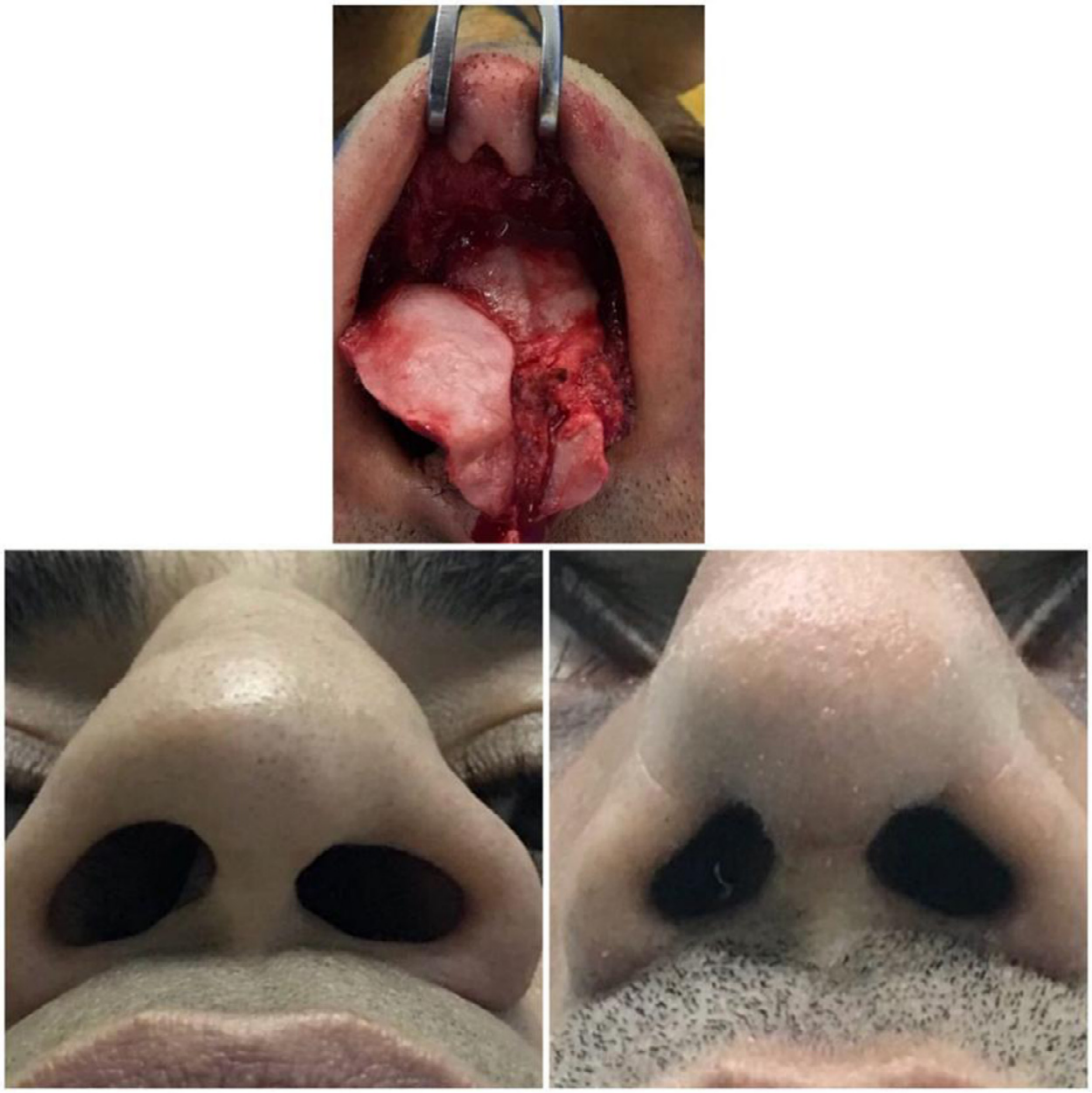
Case No. (4): Upper; preoperative finding, Lower left; Preoperative finding, Lower right; postoperative result.

Isolated nasal deformities are either overt visible deformities or occult deformities like the ones described in this paper^1^. Embryological development of the nose occurs between the 3rd and 10th week of gestation^4^. It is formed from the fusion of the medial and lateral nasal processes where the medial crus is made from the former and the lateral crus from the later. The defect may occur before the 7th week of gestation due to factors that affect the migration, proliferation or differentiation of neural crest cells, or after the 7th week of gestation due to pressure or vascular events.^1^, ^3^^,^^6^, ^7^ Cosins and Daniel^1^ had devised a nice classification, which is adopted in this paper and according to their classification; all our cases will fall in to segmental-loss category. Different sources of cartilage were used by different authors, such as lower lateral cartilage,^1^ hump cartilage.^8^ Septal cartilage graft was used in these cases, it was found practical and successful (Figures 1–4).

## Conclusion

In this series, the incidence of this deformity was about 0.016 of consecutive series of primary septorhinoplasty patients. It deserves the attention to be diagnosed and prepared for preoperatively.

## Disclosure

The author has nothing to disclose.

## Patients consent

All patient had given an informed written consent for publishing their data.
